# MicroRNAs in regulation of triple-negative breast cancer progression

**DOI:** 10.1007/s00432-018-2689-2

**Published:** 2018-06-19

**Authors:** Dominika Piasecka, Marcin Braun, Radzislaw Kordek, Rafal Sadej, Hanna Romanska

**Affiliations:** 1 Department of Pathology, Medical University of Lodz, Lodz, Poland; 2 Postgraduate School for Molecular Medicine, Medical University of Warsaw, Warsaw, Poland; 3 Department of Molecular Enzymology, Intercollegiate Faculty of Biotechnology, University of Gdansk and Medical University of Gdansk, Gdansk, Poland

**Keywords:** Triple-negative breast carcinoma, MicroRNA, EMT, CSC, Invasion

## Abstract

**Purpose:**

Dysregulation of miRNA profile has been associated with a broad spectrum of cellular processes underlying progression of various human malignancies. Increasing evidence suggests that specific microRNA clusters might be of clinical utility, especially in triple-negative breast carcinoma (TNBC), devoid of both predictive markers and potential therapeutic targets. Here we provide a comprehensive review of the existing data on microRNAs in TNBC, their molecular targets, a putative role in invasive progression with a particular emphasis on the epithelial-to-mesenchymal transition (EMT) and acquisition of stem-cell properties (CSC), regarded both as prerequisites for metastasis, and significance for therapy.

**Methods:**

PubMed and Medline databases were systematically searched for the relevant literature. 121 articles have been selected and thoroughly analysed.

**Results:**

Several miRNAs associated with EMT/CSC and invasion were identified as significantly (1) upregulated: miR-10b, miR-21, miR-29, miR-9, miR-221/222, miR-373 or (2) downregulated: miR-145, miR-199a-5p, miR-200 family, miR-203, miR-205 in TNBC. Dysregulation of miR-10b, miR-21, miR-29, miR-145, miR-200 family, miR-203, miR-221/222 was reported of prognostic value in TNBC patients.

**Conclusion:**

Available data suggest that specific microRNA clusters might play an important role in biology of TNBC, understanding of which should assist disease prognostication and therapy.

## Introduction

Despite continuous advances in early detection and development of personalized therapy, breast cancer (BCa) is still the leading cause of death from cancer among women, with age-standardized mortality rate of 12.9/100,000 worldwide (Ferlay et al. 2014; Tao et al. 2015). Comprehensive gene expression profiling has identified five major BCa molecular subtypes (luminal A, luminal B, HER2-type, triple-negative and normal-like BCa) characterised by specific morphological patterns and distinct biological properties and, more importantly, different clinical course and prognosis (Perou et al. 2000; Polyak 2007). The most aggressive, triple-negative breast cancer (TNBC), lacks expression of valid predictive markers [oestrogen receptor (ER), progesterone receptor (PR) and epidermal growth factor receptor 2 (HER2)], and thus devoid of clear therapeutic targets, it presents a serious clinical challenge. Patients with TNBC do not benefit from endocrine or HER2-targeted therapies and have worse outcome after chemotherapies in comparison to sufferers from other BCa subtypes (Lehmann and Pietenpol 2015). Shortened disease-free and overall survival of TNBC patients calls for urgent identification of new molecular targets that may improve prognostication and, above all, assist in development of efficient specific therapies.

MicroRNAs (miRNAs) are small, non-coding, endogenous, universal RNA regulators of key biological processes (Lin and Gregory 2015). In cancer, dysregulation of miRNA profile has been associated with mechanisms of disease development, including activation of invasiveness and metastasis (Lin and Gregory 2015). In TNBC, in particular, emerging in vitro and in vivo studies identified various miRNAs likely to be linked to the aggressive phenotype (Chang et al. 2015; Zhu et al. 2017; Lü et al. 2017; Paszek et al. 2017; Avery-Kiejda et al. 2017).

As metastatic process is considered the major cause of cancer-related death, our review focuses on key microRNAs of potential clinical value in TNBC, involved in regulation of main mechanisms underlying invasive progression, particularly, the epithelial-to-mesenchymal transition (EMT) and acquisition of stem cell-like properties (CSC).

## Triple-negative breast cancer—molecular features

Systemic investigation of gene expression patterns in human breast tumours revealed the molecular taxonomy of BCa dividing them into five subgroups dependent on genetic and biological similarities (Perou et al. 2000). The microarray analyses identified the triple-negative breast cancer (ER-, PR- and HER2-negative) as a clinically heterogeneous malignancy and the most aggressive BCa subtype that is characterised by high rates of tumour recurrence and poor overall survival. Aggressive phenotype of TNBC defined by poor disease-free survival, high recurrence rate and shortened time of overall survival is connected with biological and clinical factors, including high nuclear grade, high histological grade, high genomic instability, loss of suppressor genes, as well as gain of migratory, invasive and stem cell-like properties of cancer cells (Arpino et al. 2015).

## MicroRNAs

### Biology of miRNAs

MicroRNAs comprise a class of 22–25 nucleotides long, non-coding, endogenous RNA molecules, which play important regulatory roles by targeting mRNA transcripts, leading to their translational repression or degradation (Lin and Gregory 2015). Biogenesis of miRNA is under tight spatial and temporal control and is cell- and tissue-specific (Lin and Gregory 2015). In human, majority of miRNAs are encoded among introns, however, their presence was also observed in exonic regions. Production of miRNAs starts when so-called pri-miRNAs are transcribed by RNA polymerase II/III. Clustered pri-miRNAs are further converted into pre-miRNAs by a microprocessor complex consisting of RNAse III enzyme Drosha and DGCR8 (DiGeorge Critical Region 8) protein (Lee et al. 2002, 2003). Drosha cleaves 11 bp away from the single-stranded stem loop junction, converting pri-miRNA into pre-miRNA, which contains 5′ phosphate group and 2–3 nucleotides 3′ overhang (Lee et al. 2002, 2003). Pre-miRNA is translocated to the cytoplasm and cleaved by a specific endonuclease—RNAse III Dicer to finally form a single-stranded, mature miRNA (Hutvágner et al. 2001). This is then activated by the Argonaute family protein and coupled into the microRNA-induced silencing complex (miRISC), which attaches to the 3′ untranslated region (3′ UTR) of the target mRNA (Fire et al. 1998; Hannon et al. 2000; Martinez et al. 2002). The degree of complementarity between miRNA and its target mRNA determines efficacy of miRNA action. While a perfect match leads to mRNA deadenylation, and consequently its degradation, an imperfect pairing only inhibits translation of the target mRNA.

### Regulatory role of miRNAs

MicroRNAs regulate diverse cellular processes (Hwang and Mendell 2006; Shivdasani 2006; Olivieri et al. 2013), and thus create a characteristic signature/profile reflecting both tissue-specificity and developmental stage (differentiation) of the cell. For example, expression changes of specific miRNA clusters are highly informative and may be used to identify the tissue of origin of poorly differentiated tumour (Hwang and Mendell 2006). In TNBC, various miRNAs have been associated with processes essential to disease progression, such as epithelial-to-mesenchymal transition (EMT), acquisition of stem-like properties by cancer cells, migration, invasiveness, and metastatic spread.

#### Epithelial-to-mesenchymal transition

EMT, regarded as a prerequisite for metastasis, is a cellular reprogramming mechanism crucial to the ‘invasive makeover’ of cancer cells (Sethi et al. 2011; Seton-Rogers 2016; Felipe Lima et al. 2016). A fundamental event in EMT, marking the onset of the process, is the loss of E-cadherin expression, which in normal epithelial cells is required for maintenance of integrity of the entire cadherin–catenin–actin network. Regulation of E-cadherin expression at the transcriptional level is not fully understood yet, but several transcription factors, such as SNAI1/Snail1, SLUG, ZEB1, ZEB2, E47 and KLF8 (Kruppel-like factor 8) have been shown to bind to the E-cadherin promoter and repress directly its transcription (Singh and Settleman 2010; Lamouille et al. 2014; Seton-Rogers 2016; Felipe Lima et al. 2016).

The epithelial–mesenchymal switch involves changes in several pathways, including TGF-β, WNT, HIF1/2, NOTCH, NF-κB and RAS-ERK1/2. In the TGF-β pathway, the signal is generated from the TGF-β-activated kinase receptors (TGF-βRI and TGF-βRII) and processed downwards by the SMAD-dependent (formation of the activated complex of SMAD2, SMAD3 and SMAD4) or SMAD-independent (including PI3K/AKT and ERK/MAPK) signalling. This results in overexpression of SNAIL1/2 and ZEB1/2 and, finally, in the repression of E-cadherin expression (Shirakihara et al. 2011; Massagué 2012; Weiss and Attisano 2013). Activation of the WNT pathway, found particular significance in breast cancer, results in the stabilization of β-catenin, leading to the subsequent break of its interaction with E-cadherin and translocation into nucleus, where it participates in the induction of the mesenchymal-specific gene profile (Yook et al. 2006; Niehrs 2012; Lamouille et al. 2014). Notch signalling induces EMT both directly and via the crosstalk with other signalling pathways. Direct signals created by binding of Delta or Jagged family ligands to the Notch receptors cause cleavage of Notch intracellular domain (NICD), which migrates to the nucleus, where it promotes expression of SNAIL1/2 (Kaidi et al. 2007; Sahlgren et al. 2008; Wu et al. 2009; Espinoza et al. 2013). In Hedgehog (Hh) signalling, glioma 1-associated protein (GLI1), the Hh effector, promotes SNAIL1/2 expression (Kalluri and Weinberg 2009; Thiery et al. 2009). EMT can also be triggered by the signals induced by growth factor (FGF, EGF, HGF, VEGF) receptors involved in activation of the RAS-ERK1/2 or PI3K-Akt pathways. The EMT process is also strongly influenced by stimuli derived from tumour microenvironment, such as inflammation, hypoxia and metabolic or oncogenic stress (Kalluri and Weinberg 2009; Yuan et al. 2012). The existence of a positive feedback loop between pro-inflammatory microenvironment and EMT has been shown in several experiments (Mantovani et al. 2008; López-Novoa and Nieto 2009). Cohen et al. reported that inflammatory cytokines induced EMT in inflammatory breast cancer cell lines (SUM149PT, KPL4, IBC-3, SUM190PT), whereas their inhibitors blocked the process (Cohen et al. 2015). Stimulation of normal breast epithelial cells with inflammatory cytokines, such as tumour necrosis factor alpha (TNF-α) and interleukin 1beta (Il-1β), resulted in induction of EMT (mainly by upregulation of ZEB-1 and SNAIL expression), and this was associated with acquirement of invasive phenotype by the cells (Leibovich-Rivkin et al. 2013) Reversely, overexpression of the key EMT transcription factors led to increased secretion of pro-inflammatory cytokines IL-1, IL-6, IL8 by the cells, further stimulating EMT (Ricciardi et al. 2015). Decrease of reactive oxygen species caused by tumour-related hypoxia upregulated hypoxia-inducible factors (HIF1/2), which led to EMT via overexpression of ZEB1/2 and protection of SNAIL from degradation (Wang and Zhou 2011; Lamouille et al. 2013).

**Fig. 1:**
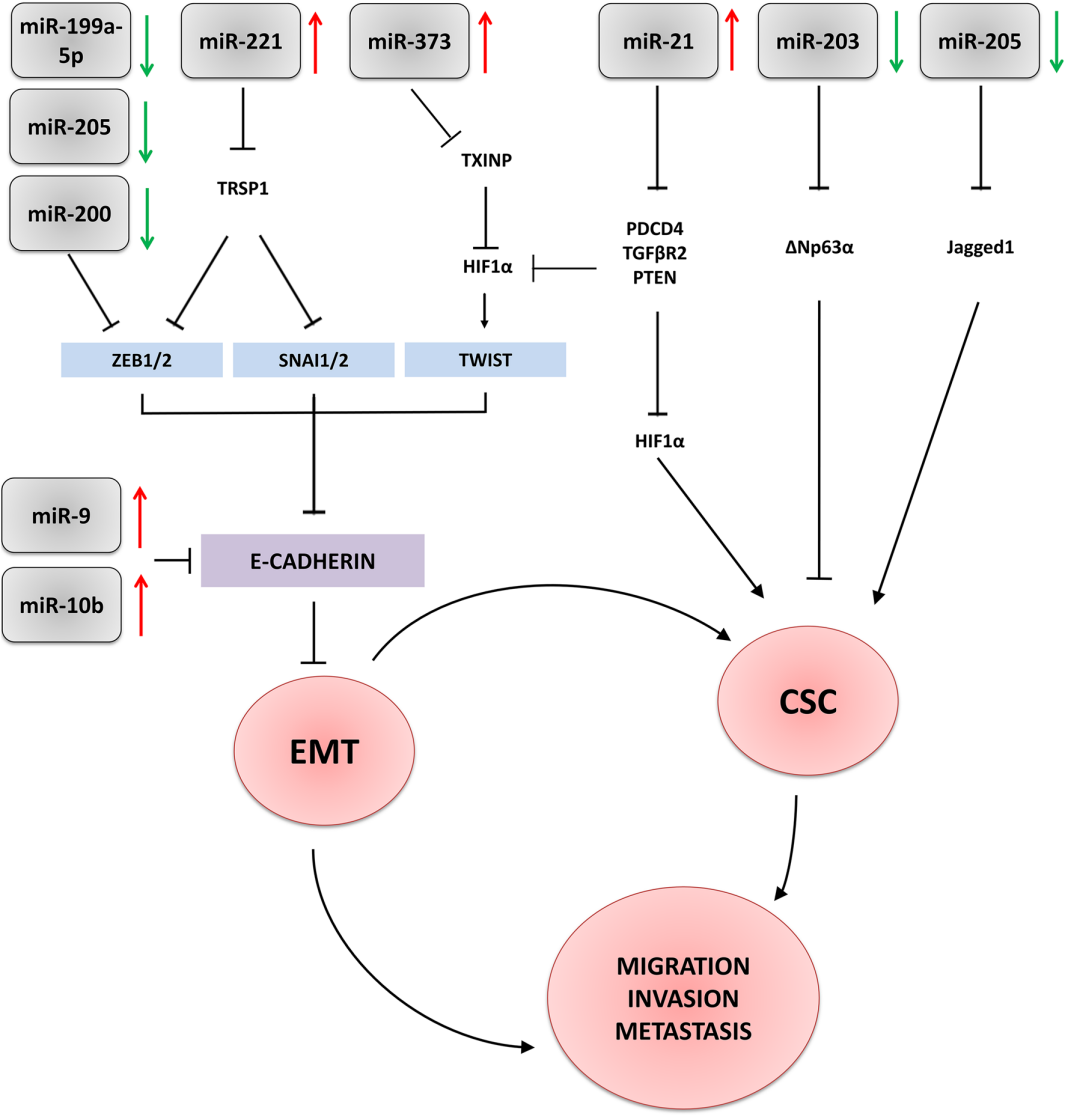
Schematic presentation of microRNAs involvement in regulation of epithelial-to mesenchymal transition (EMT) and acquisition of stem cell-like properties (CSC) in triple-negative breast carcinoma. Red/green arrows—upregulated/downregulated microRNAs; black arrows/truncated lines—activation (upregulation)/repression (inhibition) of signalling pathways

*MiRNA-200* family, which embraces miR-200a, miR-200b, miR-200c, miR-141 and miR-429, are well-known negative regulators of EMT, specifically targeting ZEB1/2 (Table 1; Fig. 1) (Korpal et al. 2008; Kalluri and Weinberg 2009; Wang et al. 2013, 2014; Humphries et al. 2014; Zaravinos and Apostolos 2015). Functional in vitro studies identified miR-200 family as downregulated in TNBC cells and confirmed their tumour-suppressive action in normal tissues (Korpal et al. 2008; Mekala et al. 2018). Humphries and colleagues showed significantly lower levels of miR-200 family members in metastatic TNBCs in comparison to other subtypes of breast cancer (Humphries et al. 2014). MiRNA-200 family contributes to the pathogenesis of TNBC via various pathways, including BRCA1/2, however, their most significant impact is exerted via regulation of EMT (Korpal et al. 2008; Humphries et al. 2014; Erturk et al. 2015). The interdependence between miRs-200 family and EMT was reported by Korpal and colleagues, who in NMuMG cells (murine mammary epithelial cells—a model of TGFβ1-induced EMT) observed a strong downregulation of all miR-200 family members (except for miR-141) upon stimulation with TGF (Korpal et al. 2008). Moreover, overexpression of miR-200 resulted in EMT repression in NMuMG cells. MiR-200 was linked to an increase of E-cadherin level and reversal of mesenchymal phenotype in 4TO7 cells, the murine TNBC cell line (Korpal et al. 2008). Gregory and co-workers obtained consistent results and found that miR-200 level was markedly lower in cells undergone EMT in response to the TGFβ treatment (Gregory et al. 2008) and inhibition of miR-200 was sufficient for induction of EMT via upregulation of *ZEB1*. Decreased expression of miR-200 family was detected in mesenchymal-like TNBC invasive human BCa cell lines (MDA-MB-435, BT-549) (Gregory et al. 2008). Overexpression of miR-200c in TNBC cells (MDA-MB-231 cell line) resulted in loss of the elongated shape associated with a motile, mesenchymal phenotype and acquisition of the epithelial-like morphology. Downregulation of miR-200b was found crucial in increase of EMT in TNBC cells by targeting ZEB1/2 and suppressing PKCα (Kolacinska et al. 2014; Humphries et al. 2014; Rhodes et al. 2015). Loss of the actin-based structure was orchestrated by miR-200c, which directly targeted actin regulatory proteins, FHOD1 and PPM1F, in a ZEB1/2-independent manner and led to the inhibition of migration and invasion of the cells (Jurmeister et al. 2012).

**Table 1:** miRNAs associated with TNBC biology and disease outcome

microRNA	Gene target	Expression in TNBC	Function in vitro	Expression in cancerous tissue and prognostic role
miR-10b	*HOXD10*	Up regulated	Promotion of cell migration and invasion (Ma et al. 2007; Edmonds et al. 2009; Han et al. 2014)	Association with lymph node metastases (Ma et al. 2007; Ouyang et al. 2014; Fkih et al. 2017)
miR-21	*PDCD4, PTEN, HIF1α, TIMP3, TM1*	Up regulated	Promotion of cell migration and invasion (Lu et al. 2008; Qi et al. 2009; Huang et al. 2009; Han et al. 2012; MacKenzie et al. 2014; Mattos-Arruda et al. 2015)	Association with poor prognosis (poor relapse-free survival) (Lu et al. 2008; Qi et al. 2009; Huang et al. 2009; Han et al. 2012; Dong et al. 2014; Medimegh et al. 2014; MacKenzie et al. 2014; Mattos-Arruda et al. 2015)
miR-29	*TTP*	Up regulated	Induction of metastasis (Drago-Ferrante et al. 2017)	No significant prognostic association (Drago-Ferrante et al. 2017)
miR-9	*CHN1*	Up regulated	Inhibition of EMT (Ma et al. 2010; Baroni et al. 2016; D’Ippolito et al. 2016; Jang et al. 2017)	Association with worse disease-free survival (Ma et al. 2010; Baroni et al. 2016; D’Ippolito et al. 2016; Jang et al. 2017)
miR-145	*MUC1, JAMA-A, ARF6*	Down regulated	Inhibition of cell motility, enhancement of p53 activity (Sachdeva and Mo 2010; Götte et al. 2010; Eades et al. 2015)	No significant prognostic association (Radojicic et al. 2011)
miR-199a-5p	*CDH1, ZEB1, TWIST*	Down regulated	Inhibition of EMT, cell migration, invasiveness, and tumour growth in vivo (Chen et al. 2016)	Association with stage of disease (Chen et al. 2016)
miR-200 family	*ZEB1*/*2, SNAI1*/*2* *FHODC1, Jag1, Maml2, Maml3* *PIKCa*	Down regulated	Inhibition of EMT, promotion of MET, inhibition of cancer cell migration, invasion, stem-cell capacity (Gregory et al. 2008; Korpal et al. 2008; Kalluri and Weinberg 2009; Jurmeister et al. 2012; Wang et al. 2013; Humphries et al. 2014; Erturk et al. 2015; Rhodes et al. 2015; Mekala et al. 2018)	Association with increased chemoresistance, lymph node involvement (Gregory et al. 2008; Korpal et al. 2008; Jurmeister et al. 2012; Pecot et al. 2013; Hill et al. 2013; Wang et al. 2013; Kolacinska et al. 2014)
miR-203	*BM1, SOX2, KLF4*	Down regulated	Reduction of stem-cell like properties, migration, invasion and metastatic capacity (Wellner et al. 2009; DeCastro et al. 2013; Zhao et al. 2015; Fite and Gomez-Cambronero 2016)	Higher levels in circulating tumour cells, discrepant results regarding survival (Madhavan et al. 2013; Liang et al. 2016; Gomes et al. 2016)
miR-205	*ZEB1*/*2, HMGB3* *TP53*	Down regulated	Reduction of proliferation, inhibition of EMT and stemness (Gregory et al. 2008; Piovan et al. 2012; Chao et al. 2014; Huo et al. 2016)	Associated with lymph node metastases (Berber et al. 2014)
miR-221/222	*TRSP1, ADIPOR1*	Up regulated	Promotion of EMT, cell migration, tumour growth in vivo, inhibition of apoptosis (Stinson et al. 2011; Nassirpour et al. 2013; Hwang et al. 2013)	Increased chemoresistance, discrepant results regarding survival (Yang et al. 2014; Kurozumi et al. 2017)
miR-373	*CD44, HIF1α*	Up regulated	Association with more aggressive phenotype of TNBC (Huang et al. 2008; Eichelser et al. 2013; Chen et al. 2015)	No data reported

*MiR-205*, in addition to the miR-200 family, has been revealed by microarray analyses as significantly reduced in cells induced to undergo EMT (Gregory et al. 2008; Piovan et al. 2012). MiR-205 expression in mesenchymal-like BCa cells is strongly downregulated in comparison to that in cells with the epithelial phenotype. Interestingly, microRNA expression profiling has shown decreased expression of miR-205 in TNBC, suggestive of its tumour-suppressive role (Huo et al. 2016). Gregory et al. demonstrated that in MDA-MB-231, MDA-MB-435 and BT-549 cells, miR-205 suppressed ZEB1/2 and, reversely, induction of EMT via TGFβ led to decrease of miR-205 (Gregory et al. 2008). This reciprocal regulatory loop between miR-205 and ZEB1/2 transcription factors is similar to that described for the miR-200 family (Gregory et al. 2008; Chao et al. 2014).

*MiR-199a-5p* is another example of microRNA that confers tumour-suppressive role and is downregulated in TNBC. Ectopic expression of miR-199a-5p in MDA-MB-231 cells significantly altered expression pattern of EMT-related genes such as *CDH1, ZEB1* and *TWIST*, leading to the inhibition of the process. Moreover, Chen et al. demonstrated that elevated level of miR-199a-5p impaired cell motility and invasiveness as well as inhibited tumour growth in vivo (Chen et al. 2016).

*MiR-9* represents a group of microRNAs upregulated in TNBC (Table 1). MiR-9-mediated downregulation of E-cadherin leads to activation of β-catenin signalling pathway and upregulation of VEGF expression. In TNBC, miR-9 was shown to be associated with *MYC* amplification, tumour grade and metastatic status (Ma et al. 2010; Jang et al. 2017). High level of miR-9 correlated with poor disease-free survival (DFS) and distant metastasis-free survival (DMSF) (Ma et al. 2010). D’Ippolito et al. observed higher expression of miR-9 in TNBC in comparison to the luminal and HER2-enriched BCa subtypes. Moreover, upon ligand activation of PDGFRβ signalling, miR-9 promoted formation of vascular-like structures by TNBC cells both in vitro and in vivo, whereas inhibition of miR-9 expression strongly decreased the number of vascular lacunae (D’Ippolito et al. 2016). Interestingly, miR-9 may also act as an important player in the cross-talk between tumour and its stroma, as exosome-mediated delivery of miR-9 was shown to induce cancer-associated fibroblast properties in normal human mammary fibroblasts (Baroni et al. 2016).

*MiR-221/222*, reported to be overexpressed in TNBC, is involved in yet another mechanism of EMT regulation (Table 1) (Stinson et al. 2011). As demonstrated by Stinson et al., miR-221 and its direct target, *TRSP1*, repress ZEB2 expression leading to E-cadherin downregulation (Stinson et al. 2011). Depletion of adiponectin receptor 1 (ADIPOR1), another miR-221’s target, induced EMT in MCF10A cells, activated NFκB and JAK2/STAT3 signalling pathways as well as increased cell migration and invasion (Hwang et al. 2013). Knockdown of miR-221 blocked cell cycle progression, promoted cell apoptosis and inhibited in vitro proliferation and tumour in vivo growth. Silencing of miR-221 increased also expression of E-cadherin, and decreased SLUG and Snail level in TNBC cell lines, such as MDA-MB-231, BT-20, and MDA-MB-468 (Nassirpour et al. 2013).

#### Cancer stem cells (CSC)

Reactivation of the stem cell programme is a phenomenon closely associated with sustained cancer progression as well as failure of eradicating therapies. By generating pools of self-renewing cancer stem cells (CSCs), primary and metastatic tumours, especially of the most aggressive type as TNBC, become more resistant to chemo- and radiotherapy (Scheel and Weinberg 2012; Hollier et al. 2013; Lim et al. 2013; Wang et al. 2015). The interdependence between EMT and breast cancer CSCs (BCSCs) was shown in various in vitro and animal models, confirming a well-documented direct link between the EMT and acquisition of stem cell-like properties (Shostak and Chariot 2011; Yamamoto et al. 2013; D’Angelo et al. 2015; Jang et al. 2015). Many signalling pathways implied in the induction of EMT, such as Wnt, Notch, TNFα, NFκB or TGFβ, control also CSCs functions (Shostak and Chariot 2011; Yamamoto et al. 2013; D’Angelo et al. 2015; Jang et al. 2015). Similarly, microRNAs involved in regulation of EMT, contribute to induction and maintenance of stemness as well as influence CSCs response to the EMT-related signals (Fig. 1).

*MiR-203* is one of the well described microRNAs, which are involved in both stemness and EMT in TNBC. In normal breast epithelial cells, miR-203 is correlated with cell differentiation by targeting ΔNp63α, the predominant TP63 isoform in mammary epithelia, vital to the maintenance of epithelial stemness. Overexpression of miR-203 induced reversal of EMT, the mesenchymal-to-epithelial transition (MET), and led to decreased proliferation and colony formation of MDA-MB-231 cells (DeCastro et al. 2013). Moreover, Wang et al. observed that upregulation of miR-203 in TNBC cell lines (MDA-MB-468, MDA-MB-231) resulted in growth and invasion inhibition, enhancement of cell differentiation and reduction of cell metastatic capacity (DeCastro et al. 2013; Zhao et al. 2015). Taube et al. showed that miR-203 was repressed via epigenetic modification (DNA methylation) to a greater degree in TNBC cells (MDA-MB-231, SUM-159) than in more differentiated luminal BCa cell lines (MCF7, T47D) (Zhao et al. 2015; Fite and Gomez-Cambronero 2016). Studies by Wellner and colleagues showed that miR-203 is under control of ZEB1, which acts as both an inducer of the TGFβ-related EMT as well as a mediator of differentiation and self-renewal of CSC. Thus, by repressing stemness-inhibiting microRNAs, i.e. miR-200, miR-203 and miR-183, ZEB1 promotes tumorigenicity of the cells (Wellner et al. 2009).

*MiR-205* is one of the critical regulator of stemness, also in breast cancer cells. Its physiological role is to supress ZEB1/2 expression, preventing EMT processes and maintaining differentiated state of cells. MiR-205 is repressed by the ligand Jagged1, a stroma-derived factor, promoting cancer stem cell-like phenotype (Lu et al. 2013; Chao et al. 2014). Silencing of miR-205 in mammary epithelial cells stimulated EMT, disrupted epithelial cell polarity and expanded stem cell population (Lu et al. 2013; Chao et al. 2014). Interestingly, in vivo studies indicated that miR-205-deficient mice spontaneously developed mammary lesions, while activation of miR-205 markedly diminished breast cancer cell stemness (Bojmar et al. 2013; Chao et al. 2014). In TNBC, downregulation of miR-205 resulted in chemoresistance, mainly due to induction of EMT and stemness (Sempere et al. 2007).

*MiR-21* overexpression, identified in many solid tumours, is best characterised in TNBC (Table 1). Existing evidence demonstrates that TGFβ stimulation increases miR-21 expression in cancer cells, which in turn upregulates EMT process. This is associated with induction of BCSC-like phenotype and increase of hypoxia-inducible factor (HIF1α) levels. MiR-21 targets many different gene transcripts, such as PDCD4, PTEN, HIF1α, TIMP3 or TM1 mRNAs (Table 1) (Lu et al. 2008; Qi et al. 2009; Huang et al. 2009; Han et al. 2012; Mattos-Arruda et al. 2015). Han et al. observed that breast cancer stem cells undergone EMT express higher miR-21 levels than BCa cells not subjected to EMT. Interestingly, downregulation of miR-21 in BCSCs leads to MET, decrease of HIF1α and suppression of cell migration and invasion (Han et al. 2012). Although a direct association between miR-21 and EMT requires more thorough investigation, this microRNA seems to be of particular importance for BCa pathophysiology and may serve as a good indicator of treatment efficacy. Interestingly, in addition to its presumptive function in TNBC cells, high miR-21 level in tumour stroma was found to be also associated with poor disease outcome of the patients (MacKenzie et al. 2014).

#### Migration, invasion and metastasis

*MiR-145* is representative of microRNAs that regulate cells migration, invasion, and metastasis (Table 1). In BCa cells, it targets mucin-1 (MUC1) and c-MYC—mRNA, both associated with cell invasiveness (Sachdeva and Mo 2010). MiR-145 downregulation is detected in approximately 10% of invasive breast carcinomas. MiR-145 was also shown to regulate invasion in TNBC by regulating ARF6 protein (Eades et al. 2015). Recent studies indicate that upregulation of miR-145 significantly reduces cell motility in MDA-MB-231 cells via targeting junctional adhesion molecule A (JAMA-A) and fascin as well as through effect on expression pattern of several motility-related proteins such as ROCK1, FSCN1 or TRMP3 involved in regulation of actin stress fibres or formation of filopodia (Sachdeva and Mo 2010; Götte et al. 2010).

*MiR-373* is frequently upregulated in TNBC tissue and blood serum. In contrast to miR-145, its downregulation was found to impair cell migration and invasiveness (Table 1) (Huang et al. 2008; Eichelser et al. 2013; Chen et al. 2015). MiR-373 targets transcripts of *CD44* and *TXNIP*, and activates two important EMT-inducers, HIF1α and Twist (mainly by targeting *TXINP*, identified as a metastasis suppressor), which in turn, in a positive feedback loop, upregulates miR-373 expression. Chen et al. showed that upregulation of miR-373-TXINP-HIF1α-Twist axis correlated with poor outcome of breast cancer patients. This suggests that activation of this signalling pathway may serve as both a potential biomarker and a new therapeutic target (Chen et al. 2015).

*MiR-10b* is highly expressed in TNBC cell lines (MDA-MB-231 and SUM1315), when compared to normal mammary epithelial (HMECS, MCF10A) or tumourigenic, but non-metastatic cells (SUM149 or SUM159), and enhances metastatic potential of cells grown in xenografts (Ma et al. 2007; Edmonds et al. 2009). MiR-10b positively regulates cell migration and invasion as well as influences expression of miR-9 (Table 1). High level of TGFβ was associated with upregulation of miR-10b in TNBC cell lines, whereas inhibition of miR-10b partially reversed EMT, and suppressed cell motility and proliferation (Han et al. 2014). MiR-10b was also reported to be positively correlated with Twist and was considered as an important mediator of twist-induced motility and invasiveness (Ma et al. 2007).

### Prognostic value of miRNAs in TNBC

Despite many attempts in development of personalized therapy among molecular subtypes of TNBC, no breakthrough has been achieved yet. The guidelines for treatment of TNBC patients still encompass conventional surgery, radiotherapy, and chemotherapy (individually or in combination) (Costa and Gradishar 2017). Although some reports suggest that early response to specific chemotherapeutic regimens of TNBC is better than other BCa subtypes, TNBC patients are doomed to poor prognosis and chemoresistance (Pareja et al. 2016). The pattern of several microRNAs is substantially altered in TNBC suggesting they are likely to serve as useful prognostic factors in the disease (Dong et al. 2014; Sahlberg et al. 2015; Liu et al. 2017; Lü et al. 2017). For instance, decreased expression of miR-155 predicted poor overall survival in TNBC patients, while elevated levels of miR-21, miR-27a/b, miR-210, and miR-454 were associated with shorter overall survival (Medimegh et al. 2014; Sahlberg et al. 2015; Thakur et al. 2016; Lü et al. 2017). Similarly, decreased expression of miR-374a/b and increased level of miR-454 correlated with shorter disease-free survival (Radojicic et al. 2011).

Other panels of miRNAs was identified to be associated with chemoresistance (Gasparini et al. 2014; Ouyang et al. 2014; Shen et al. 2014; Sahlberg et al. 2015). For example, expression of miR-181a was elevated in TNBC tissue samples from patients who did not respond to neo-adjuvant chemotherapy and was significantly inversely correlated with chemo-sensitivity (Ouyang et al. 2014). In vitro studies by Ouyang et al. demonstrated that in the MDA-MB-231 cell line as well as in TNBC tissue samples, upregulation of miR-155-5p, miR-21-3p, miR-181a-5p, miR-181b-5p, miR-183-5p and downregulation of miR-10b-5p, miR-451a, miR-125b-5p, miR-31-5p, miR-195-5p were associated with chemoresistance to doxorubicin (Korpal et al. 2008; Chen et al. 2012; Kong et al. 2014; Liu et al. 2015; Fkih et al. 2017). Low level of miR-200c was shown to be connected with resistance to doxorubicin, poor response to radiotherapy and elevated expression of multidrug resistance gene (Korpal et al. 2008). Identification of miRNA clusters, whose deregulated levels accompany resistance to chemotherapy, may open new avenues in development of more efficient therapies.

## Conclusions

This review focused on several microRNAs shown to be specific to triple-negative breast carcinoma. Their role in TNBC biology was discussed in relation to molecular processes underlying disease progression, with particular emphasis on the epithelial-to-mesenchymal transition. Although, for the most part, the degree of involvement in TNBC pathophysiology remains to be established, increasing evidence suggests that specific microRNA clusters might be of clinical utility as both predictive markers and potential therapeutic targets in this highly aggressive form of breast cancer.
